# Basic Measures of Auditory Perception in Children: No Evidence for Mediation by Auditory Working Memory Capacity

**DOI:** 10.3389/fnhum.2020.591101

**Published:** 2020-11-12

**Authors:** Srikanta K. Mishra, Udit Saxena

**Affiliations:** ^1^Department of Communication Sciences & Disorders, The University of Texas Rio Grande Valley, Edinburg, TX, United States; ^2^MAA Institute of Speech & Hearing, Hyderabad, India

**Keywords:** auditory memory, auditory processing, auditory development, digit span, frequency discrimination, gap detection, temporal resolution

## Abstract

Immature auditory perception in children has generally been ascribed to deficiencies in cognitive factors, such as working memory and inattention. This notion appears to be commonly accepted for all children despite limited empirical evidence. In the present work, we examined whether working memory capacity would predict basic aspects of hearing, pure-tone frequency discrimination and temporal gap detection, in typically-developing, normal-hearing children (7–12 years). Contrary to our expectation, working memory capacity, as measured by digit spans, or intrinsic auditory attention (on- and off-task response variability) did not consistently predict the individual variability in auditory perception. Present results provide no evidence for a role of working memory capacity in basic measures of auditory perception in children. This lack of a relationship may partly explain why some children with perceptual deficits despite normal audiograms (commonly referred to as auditory processing disorders) may have typical cognitive abilities.

## Introduction

Many aspects of auditory perception in children follow a protracted period of development (Sanes and Woolley, 2011). This developmental period parallels the development of working memory—briefly, a limited capacity cognitive system to store and manipulate information (Baddeley, 2012). Generally, working memory increases steeply up to 8 years of age, and shows more gradual improvement thereafter until about 11–12 years of age (e.g., Gathercole, 1999). Due to the sequential and temporal nature of sound, working memory appears to be important for auditory processing. It is intuitively appealing to explain the immature auditory performance in children in terms of cognitive factors such as working memory capacity.

In recent years, it is thought that working memory may contribute to the variability in auditory perception among children. In a population study, Moore et al. (2010) showed that poor performance on auditory tasks, e.g., frequency discrimination, in children (6–11 years) is attributable to cognitive abilities such as attention and working memory. In contrast, other studies have failed to find a relationship between measures of working memory and performance on auditory tasks, such as frequency discrimination and gap detection, for children with suspected auditory processing disorders (Sharma et al., 2009; Ahmmed et al., 2014; Tomlin et al., 2015). Table 1 presents a summary of relevant findings and demonstrates some inconsistencies in the literature. It is currently unclear whether a relationship between working memory and auditory perception exists for typically-developing children without listening difficulties.

**Table 1:** Partial correlations between auditory and working memory measures.

	**Forward digit span**	**Backward digit span**	**Test-retest FD**	**Test-retest GD**
Frequency discrimination	0.14 (−0.21^*^^b^; 0.14^ns^^,^ ^c^)	0.14	0.16^†^	0.04
Gap detection	0.15 (−0.20^ns^^,^ ^a^; 0.11^ns^^,^ ^d^)	−0.12 (0.06^ns^^,^ ^d^)	0.02	0.08

*One-tailed tests (n = 26); previous findings are in the parenthesis*.

a *Sharma et al. (2009): suspected auditory processing disorder (7–12 years)*.

b *Moore et al. (2010): population study (6–11 years), reported for digit span*.

c *Ahmmed et al. (2014); suspected auditory processing disorder (6–11 years), reported for digit span*.

d *Tomlin et al. (2015): suspected auditory processing disorder and controls (7–12 years)*.

* p < 0.001;

† *r = 0.42, p = 0.013 without controlling for age; ns, not significant*.

The extent to which auditory perception in children can be attributed to cognitive factors has direct and important translational implications for defining auditory processing disorders—a longstanding, controversial as well as a contemporary issue in pediatric audiology and auditory neuroscience (Cacace and McFarland, 2013; Moore et al., 2013; Moore, 2018; Wilson, 2018). For example, the lack of knowledge about the influence of cognitive influences on auditory perception tests could lead to a speculative assumption that cognitive ability is causally related to poor auditory performance in children. This, in turn, could result in the misdiagnosis of true auditory disorders (Keith et al., 2018).

In the current study, we examined if interindividual variation in basic measures of auditory perception can be explained by working memory capacity in typically-developing children. This rested on the hypothesis that working memory is related to auditory perception in children (Moore et al., 2010). Working memory and attention interact closely during the encoding and manipulation of information (Chun and Turk-Browne, 2007). Moore et al. (2010) reported variable test-retest results for children with poor auditory performance and attributed it to fluctuations in attention, termed as “intrinsic attention.” An additional goal of the current study was to determine the influence of intrinsic auditory attention, defined as the response variability in a given listener on the psychoacoustic performance in children. Working memory was assayed using the digit span—the most common, easy-to-administer measure of verbal short-term memory in children. The forward digit span represents the phonological loop, whereas the backward digit span involves the manipulation of information while storing the information in the immediate memory and thus, represents a complex memory span associated with both the central executive and the phonological loop (Gathercole, 1999). For measures of auditory perception, we specifically focused on two basic aspects of hearing: frequency discrimination (FD) and temporal resolution, that have different developmental trajectories. The ability to hear a change in the frequency of a pure tone in children matures between 8 and 12 years of age (Sutcliffe and Bishop, 2005; Moore et al., 2010, 2011; Buss et al., 2014). In contrast, temporal resolution—the ability to detect a rapid change in a sound over time—as measured by gap detection (GD) is mature relatively early in childhood by 5–6 years of age (Werner, 2010; Werner and Leibold, 2011). Overall, the present study aimed to define the role of auditory working memory in basic aspects of hearing for children.

## Methods

### Listeners

Data were collected from typically-developing children (*n* = 30) sub-grouped into younger (7–9 years; *n* = 17; females = 10) and older children (10–12 years; *n* = 13; females = 7) and young adults (*n* = 29; 19–26 years; mean = 20 years; 17 females). All listeners had normal hearing thresholds (≤15 dB HL) at standard octave frequencies and normal A-type tympanograms (Jerger, 1970). Children had no signs of any developmental disorder, as determined by a standard case history questionnaire. The auditory and working memory tests were randomized in order between listeners and were conducted in a sound-booth. Each test session lasted for 2.5–3 h.

### Auditory Tests

Psychophysical tasks were implemented using the System for testing auditory responses via child-friendly computer games in a three-interval, three-alternative (oddball) forced-choice paradigm (Barry et al., 2010). The listener's task was to respond to the interval that contained the odd sound. Familiarization preceded test runs and included actual test presentation at 70 dB SPL via TDH 39P supra-aural headphones. In addition to threshold estimates, the absolute value of the test-retest difference for each test was also obtained. Test-retest measures were obtained with a brief break within the same measurement session.

Stimuli for frequency discrimination were 200-ms tones, raised with 10-ms cosine ramps, with an interstimulus interval of 400-ms. The frequency of the target tone varied adaptively for a total of 25 trials, initially by 50% of the standard (1,000 Hz), using 1-down,1-up rule until the first reversal after which the staircase followed 3-down, 1-up rule with a factor of √2 (Moore et al., 2008). Threshold (a percentage of the standard frequency, ΔF%), computed as the geometric mean of ΔF from the last two reversals, was averaged across two consecutive tests for a given listener.

Broadband noise (100–10,000 Hz), shaped with 1-ms cosine ramps was used for gap detection measurements. The leading marker duration was 300 ms, whereas, the trailing marker randomly varied between 250 and 350 ms. The leading and trailing markers were separated by a fixed 1-ms gap in the standard interval. The gap (intial = 20 ms) in the target interval varied adaptively similar to the frequency discrimination procedure. The threshold was defined as the geometric mean from the last two reversals and was averaged across two consecutive tests.

### Working Memory Tasks

Digit span tests were measured using an adaptive procedure (1-up/1-down) controlled via Angel Sound, a PC-based software (Mishra and Boddupally, 2018). Traditional and computerized versions of tests yield similar digit span scores (Tractenberg and Freas, 2007). Stimuli were presented via headphones at 70 dB SPL routed using an audiometer. Digits were randomly selected between “0” through “9” and were played at a rate of 1/s in an auditory-only mode, with three digits in the first trial. Listeners were allowed unlimited time to respond by clicking on a window labeled “0” through “9” on the computer screen that was visible throughout the testing. Forward digit span required listeners to respond with the original sequence of digits, whereas listeners responded in the reverse order of presentation for the backward digit span. The digit span (raw) score was the mean of correctly recalled digits from all but the first two reversals, averaged across two consecutive tests, each with 25 trials.

### Statistical Analyses

Frequency discrimination and gap detection thresholds, and absolute values for within-session test-retest differences were log-transformed for all analyses. Digit span raw scores were *z*-transformed. The effect of age group on auditory and working memory measures was tested using the one-way analysis of variance (ANOVA). For significant ANOVA, pair-wise comparisons were conducted using Bonferroni corrections. Multiple linear regression models were fitted to explain the variance in auditory measures separately for children and adults. A regression model was fitted with age (log-transformed), sex (dummy coded), audiometry threshold average (500, 1,000, and 2,000 Hz), forward and backward digit span scores, the test-retest differences for FD and GD as predictors for response variables: FD and GD thresholds, separately. Auditory thresholds were included in the model as the audibility in children with hearing impairment is associated with cognition (Moore et al., 2019). Partial correlations between auditory measures and working memory variables were examined while controlling for age effects. One-tailed tests were conducted because it was hypothesized that higher working memory capacity (higher digit span scores) would be associated with lower FD or GD thresholds. For further analysis, all children were categorized into two groups, low and high thresholds, based on median values separately for FD and GD tests. Multivariate analysis of covariance (MANCOVA) with FD and GD median-based groups and age as the covariate was conducted to examine if digit span, test-retest FD, and GD differences differ between low and high threshold groups. An effect was considered as statistically significant if *p* < 0.05.

## Results

### Developmental Effects on Working Memory and Auditory Measures

**Figure 1:**
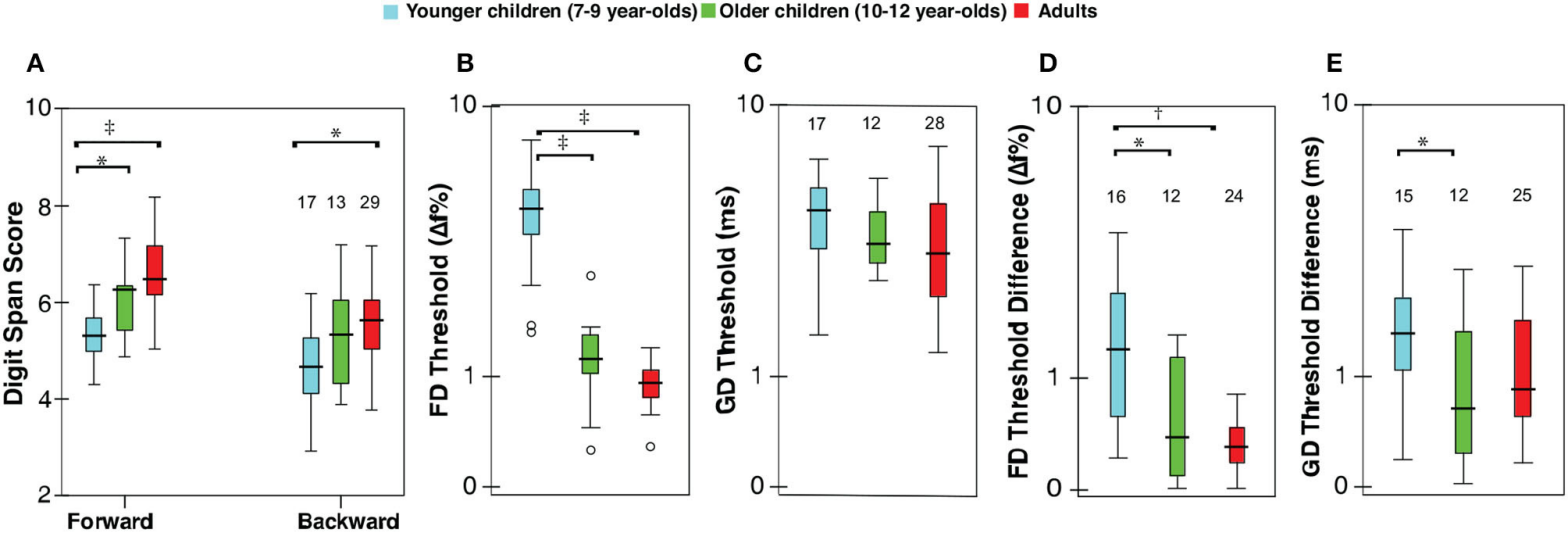
Box-Whisker plots showing **(A)** digit span scores (for forward and backward span), **(B)** frequency discrimination (FD) thresholds, expressed as a percent of the standard frequency (1,000 Hz), **(C)** gap detection (GD) thresholds (ms), test-retest differences for **(D)** FD, and **(E)** GD thresholds; ordinate is (safe^1^) log-scaled for all but digit span. Horizontal lines indicate the median, boxes span from the 25th to 75th percentile, and whiskers show the maximum and minimum values. Separate data points are outliers. The numbers on top show the included data and were same for FD and GD thresholds. Significant differences between groups are indicated at the top part of the figure (*p < 0.05, ^†^*p* < 0.01, ^‡^*p* < 0.001).

All listeners completed the working memory testing. One child (10-year-old) and one adult did not complete the auditory testing. Of those who completed auditory testing, repeated measures could not be obtained from two younger children, one older child, and six adults. Non-completion was only due to time constraints. Table 2 presents a summary of raw scores for various measures. The pure-tone hearing threshold average was not significantly different between groups (*F*_2,56_ = 0.48, *p* = 0.62). Figure 1 shows the distributions for digit span scores, thresholds, and test-retest threshold differences for FD and GD. Responses for all variables appear to improve with age, with older children exhibiting adult-like values. ANOVA showed significant effect of age group for all variables but GD thresholds (forward digits: *F*_2,55_ = 17.71, *p* < 0.001; backward digits: *F*_2,55_ = 6.60, *p* < 0.01; FD: *F*_2,54_ = 71.39, *p* < 0.001; GD: *F*_2,54_ = 1.43, *p* = 0.25; test-retest FD: *F*_2,49_ = 7.97, *p* < 0.01; test-retest GD: *F*_2,49_ = 3.30, *p* = 0.05). Significant group differences from multiple comparison analysis are marked in Figure 1. For forward digit span, the scores were lower for younger children compared with older children and adults; however, older children and adult scores were indistinguishable. Younger children scored lower on the backward digit span test than adults, whereas scores for older children were not significantly different from those of younger children or adults. The pattern of results for FD thresholds and FD test-retest differences was similar to the forward digit span. Gap detection thresholds were similar for all age groups. However, GD threshold test-retest differences were higher in younger children compared to older children but not relative to adults.

**Table 2:** Mean and standard deviations in the parenthesis for auditory and working memory measures.

	**7–9 yr-olds**	**10–12 yr-olds**	**Adults**
Pure-tone average (dB HL)	8.24 (4.66)	6.54 (5.16)	7.76 (4.74)
Frequency discrimination (ΔF%)	4.88 (2.26)	1.29 (0.64)	0.92 (0.24)
Gap detection (ms)	4.33 (1.46)	3.91 (1.06)	3.62 (1.75)
Forward digits	5.28 (0.56)	6.11 (0.86)	6.57 (0.77)
Backward digits	4.60 (0.86)	5.32 (1.05)	5.58 (0.90)
Test-retest FD (ΔF%)	1.62 (1.18)	0.67 (0.65)	0.33 (0.20)
Test-retest GD (ms)	1.75 (1.06)	0.98 (0.92)	1.13 (0.79)

### Relationship Between Working Memory and Auditory Measures

**Figure 2:**
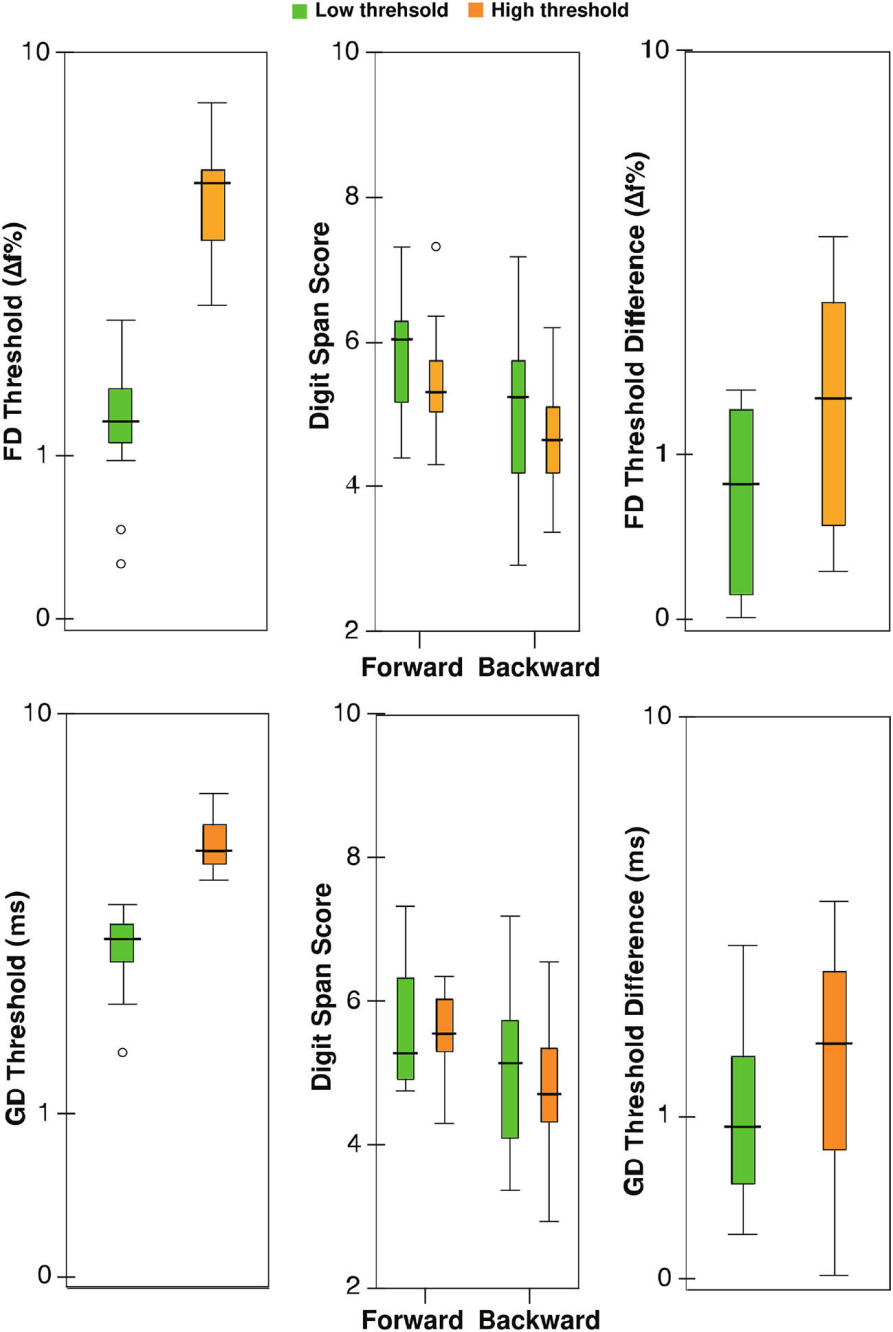
Box-Whisker plots showing thresholds, digit span scores, and test-retest differences for lower and higher (poor) threshold groups for children based on median FD (upper panel) and GD thresholds (lower panel); ordinate is (safe^1^) log-scaled for all but digit span. Horizontal lines indicate the median, boxes span from the 25th to 75th percentile, and whiskers show the maximum and minimum values. Separate data points are outliers.

For frequency discrimination in children, multiple linear regression indicated that there was a collective significant effect between the age, audiometry threshold average, sex, forward and backward digit span scores, test-retest differences for FD and GD (*F*_7,18_ = 3.18, *p* = 0.02, *R*^2^ = 0.55, *n* = 26). Only age was significant in the model (β = −3.52, *t* = −3.05, *p* = 0.007) among individual predictors. The regression model was not significant for adults (*F*_7,14_ = 0.39, *p* = 0.89, *n* = 22). For gap detection, the model was neither significant for children (*F*_7,18_ = 1.98, *p* = 0.12, *n* = 26) nor for adults (*F*_7,14_ = 0.96, *p* = 0.49, *n* = 22). For all regression analyses, the variance inflation factor was <3, suggesting a lack of multicollinearity. Table 1 shows partial correlations relating FD and GD thresholds with working memory measures for children. Figure 2 shows the digit span scores and test-retest differences for FD and GD for the median-split groups for children based on FD and GD thresholds. Note that age was not a covariate for this plot. MANCOVA revealed no statistically significant difference between the low and high threshold groups on the combined working memory variables after controlling for age (FD: *F*_4,18_ = 0.71, *p* = 0.60, Wilks' Λ = 0.86; GD: *F*_4,18_ = 0.71, *p* = 0.99, Wilks' Λ = 0.99).

## Discussion

Poor auditory performance in children is thought to be the result of immature cognitive factors (Moore, 2012; Moore et al., 2013; Magimairaj and Nagaraj, 2018). The goal of the present study was to examine the cognitive factors (specifically, working memory and intrinsic attention) that contribute to the variability in basic aspects of auditory perception in typically-developing, normal-hearing children. Working memory was assayed using forward and backward digit span tasks, whereas intrinsic attention for specific auditory tasks was indexed by test-retest threshold variability. Contrary to common predictions, the present study failed to find evidence for the explanatory power of working memory or inattention (as measured by digit spans and test-retest response variability respectively) in accounting for individual differences in basic auditory processing tasks for children. The developmental timelines for frequency discrimination and gap detection are consistent with the relevant literature (Sanes and Woolley, 2011; Werner and Leibold, 2011). FD thresholds and the response variability (test-retest) in older children (10–12 years) were adult-like. In contrast, GD thresholds in younger children (7–9 years) were mature; however, their response variability (test-retest difference) was immature (Figure 1E). This is a novel finding and may suggest residual immaturities in gap detection for younger children even though their thresholds are adult-like. The main findings were the lack of relationships between digit span scores and auditory perceptual measures (FD and GD thresholds). In addition, intrinsic auditory attention for on-the-task (i.e., FD or GD response variability) and intrinsic attention for off-the-task (i.e., FD response variability for the GD test and GD threshold variability for the FD task) failed to predict performance on basic measures of auditory perception for children. Likewise, working memory and intrinsic auditory inattention measures were indistinguishable between low- and high-threshold groups categorized based on FD and GD thresholds, separately.

One consideration is that the digit span measures may be suboptimal in reflecting working memory capacity as digits are highly practiced and have a strong relationship to language ability (e.g., Jacquemot and Scott, 2006). However, it is important to clarify that the mapping between working memory tasks and construct may not be straightforward, and there is no one or gold standard paradigm that confirms to engage the neural circuitry of working memory (Jarrold and Towse, 2006). Among several measures of working memory, the digit span task outnumbers all tasks by a factor of at least 16:1 (Jones and Macken, 2015). Using digit span and response variability measures, Moore et al. (2010) showed significant differences between children with higher and lower FD thresholds. However, this comparison was between two extreme groups: upper 95% (good) or lower 5% (poorer) thresholds. In addition, Moore et al. (2010) tested children in the school environment. For unclear reasons and surprisingly, FD thresholds are reported to be lower and less variable in school studies relative to laboratory experiments (Moore et al., 2008). Similar to the present results, Zhang et al. (2016) failed to find an association between digit span scores and FD thresholds for young adults. The relationship was evident only for a particular test paradigm that involved roving the standard frequency. We found that response variability was related to FD thresholds, but this relationship was not robust after controlling for age (Table 1). The present findings raise an important question regarding the degree to which the relationship between response variability and FD thresholds are independent of developmental age.

Without a doubt, auditory perception in children necessitates a certain degree of cognitive abilities; however, the working memory capacity or intrinsic auditory attention, despite individual variability, in typically-developing children is sufficient for age-appropriate auditory perception, similar to speech-in-noise perception in young adults (Füllgrabe and Rosen, 2016). Nevertheless, it is possible that working memory assayed by a different measure (e.g., visual working memory) may predict performance in a relatively complex auditory task (e.g., spectro-temporal ripple discrimination thresholds) for hearing-impaired children (Kirby et al., 2019). Notwithstanding the evidence, the mechanisms for such a relationship between visual memory and auditory discrimination are unclear, and at least, for simple pure-tone frequency discrimination, no similar relationship between visual inattention and auditory discrimination exists (Moore et al., 2008).

A recent study reported that children with minimal and mild degree of hearing impairments (unknown hearing loss type) have reduced auditory processing, including higher FD thresholds, and lower digit span scores (Moore et al., 2019). Although we could detect subtle developmental trends in FD and GD tests (Figure 1), a potential concern could be that larger sample sizes may reveal a significant relationship between working memory and auditory measures. However, Moore et al. (2010) detected only a weak or low (0.2) correlation, even with a sample size of 1,469 in a pediatric population study. That means only ~4% of the total variation of FD thresholds can be explained by variation in digit span scores. The practical significance, if any, of this minimal effect is unclear. One speculation is that children may become dependent on cognitive factors for auditory perception when the sensory information is compromised due to auditory disorders, e.g., hearing impairment. All things considered, the present findings suggest that individual differences in working memory cannot explain the variances observed in basic measures of auditory perception for children with normal hearing.

There has been a recent upsurge in efforts to predict auditory perception, auditory learning, and speech-language development based on working memory (e.g., Figure 1, Füllgrabe and Rosen, 2016). Attempts have also been made to relate cognitive variables, such as working memory and attention, with perceptual deficits in children with normal audiograms, commonly referred as auditory processing disorders (Sharma et al., 2009; Moore et al., 2013; Ahmmed et al., 2014; Tomlin et al., 2015; Moore, 2018). In the light of present findings, it is not surprising that evidence for a consistent relationship between working memory and perceptual deficits is lacking for children with auditory processing disorders. The present results fail to lend credence to the role of auditory working memory as a mediating factor in auditory perception for typically developing children. It may indirectly support that children with listening disorders can have typical auditory working memory.

## Data Availability Statement

The raw data supporting the conclusions of this article will be made available by the authors, without undue reservation.

## Ethics Statement

The studies involving human participants were reviewed and approved by MAA Institutional Review Board. Written informed consent to participate in this study was provided by the participants' legal guardian/next of kin for children and by adult participants.

## Author Contributions

SM conceptualized, analysed, and wrote the draft. US conceptualized, collected data, and revised the draft. All authors contributed to the article and approved the submitted version.

## Conflict of Interest

The authors declare that the research was conducted in the absence of any commercial or financial relationships that could be construed as a potential conflict of interest.

## Footnotes

^1^The safe log scale uses a modified function to handle 0. The safe log formula is sign(x) * log(1 + abs(x)). This was used only to represent the 0 in the ordinate for FD and GD thresholds and their differences. All other log transformations were done with the standard base 10 procedure.
